# TRPV1 in skeletal muscles mediates the improvement of cardiac function induced by acupuncture at the PC6 acupoint in rats with acute myocardial infarction

**DOI:** 10.1186/s13020-026-01384-2

**Published:** 2026-05-06

**Authors:** Xue Liu, Yongjian Wu, Yongxiao Zhai, Weifang Gao, Xiaoyu Meng, Wubo Pei, Yuxin Fang, Yi Guo, Yangyang Liu

**Affiliations:** 1 Research Center of Experimental Acupuncture Science of Tianjin University of Traditional Chinese Medicine, Tianjin, 301617 China; 2 School of Acupuncture-Moxibustion and Tuina of Tianjin University of Traditional Chinese Medicine, Tianjin, 301617 China; 3 National Clinical Research Center for Chinese Medicine, Tianjin, 300381 China; 4 Tianjin Key Laboratory of Modern Chinese Medicine Theory of Innovation and Application, Tianjin, 300051 China

**Keywords:** Acupuncture, Muscle layer, Mechanosensitive ion channel, TRPV1, AMI

## Abstract

**Background:**

The acupoint is the initial response site to acupuncture stimulation, and changes in the acupoint affect the subsequent therapeutic effects of acupuncture. However, the mechanism of acupuncture from this initial site has not yet been clarified. Based on an experimental model of acupuncture at Neiguan (PC6) for intervening in acute myocardial infarction (AMI), this study aimed to 1) investigate whether acupuncture-induced local muscle contraction at the PC6 acupoint mediates the therapeutic effect of acupuncture, and 2) explore the initiation mechanism of acupuncture’s effect from the perspective of transient receptor potential vanilloid 1 (TRPV1).

**Methods:**

Sprague–Dawley (SD) rats were used to establish the AMI model via ligation of the left anterior descending coronary artery (LAD). 1.To identify the key tissue layer mediating acupuncture efficacy: SD rats were randomly divided into the Sham group, AMI group, AMI + acupuncture at muscle-layer (ACU-M) group, and AMI + acupuncture at subcutaneous-layer (ACU-S) group. Cardiac function was evaluated using a small animal ultrasound imaging system; myocardial ischemic area was measured via 2,3,5-triphenyltetrazolium chloride (TTC) staining, and serum norepinephrine (NE) levels were detected using an enzyme-linked immunosorbent assay (ELISA) kit. 2.To verify the role of muscle contraction: Another cohort of SD rats was randomly divided into the Sham + Vehicle (0.9% sodium chloride, NaCl) group, AMI + Vehicle group, AMI + Vehicle + acupuncture group and AMI + succinylcholine chloride (Scc, muscle relaxant) + acupuncture group. Acupuncture was performed post-AMI modeling, with the same efficacy indexes as above. Additionally, PC6 tissue and C5–T1 segmental dorsal root ganglia (DRG) were collected from each group. Immunofluorescence (IF) staining and western blot (WB) analysis were used to detect TRPV1 expression in the acupoint muscle layer and TRPV1-positive neuron activation in DRGs. 3. To clarify the role of TRPV1: a TRPV1 inhibitor was microinjected into the PC6 muscle layer before acupuncture; changes in TRPV1 (acupoint/DRGs), cardiac function, myocardial ischemic area, and serum NE were measured to evaluate the relationship between acupuncture effect and TRPV1.

**Results:**

Both the acupuncture at muscle and subcutaneous layer improved cardiac function, reduced the area of myocardial ischemia, and lowered serum NE levels in AMI rats, however the modulatory effect of acupuncture at muscle layer was more pronounced in AMI rats. PC6 injection of Scc followed by acupuncture reversed the modulatory effect of acupuncture on AMI rats. WB and IF results both showed that compared with the AMI group, TRPV1-positive area of the muscle layer in the acupoint area, protein expression as well as activated TRPV1-positive neurons in the DRG were significantly increased, while with the use of Scc followed by acupuncture, TRPV1-positive expression in the muscle layer of the acupoint area as well as activated TRPV1-positive neurons in the DRG were significantly decreased. Muscle injection of TRPV1 inhibitors followed by acupuncture significantly reduced TRPV1-positive expression in the muscle layer of the acupoint area as well as activated TRPV1-positive neurons in the DRG compared with the muscle injection of vehicle group, and also suppressed the improvement of cardiac function and myocardial ischemic area in rats with AMI by acupuncture.

**Conclusion:**

The muscle layer at the PC6 acupoint is the primary tissue mediating the therapeutic effect of acupuncture for AMI. Acupuncture-induced muscle contraction at the acupoint serves as a key link in this effect: it upregulates TRPV1 expression in the acupoint muscle layer and activates TRPV1 ion channels in C5–T1 DRGs. Microinjection of a TRPV1 inhibitor into the acupoint muscle layer reverses acupuncture’s cardioprotective effect in AMI rats, confirming that TRPV1 in the acupoint muscle layer is a critical mediator initiating the acupuncture effect.

**Supplementary Information:**

The online version contains supplementary material available at 10.1186/s13020-026-01384-2.

## Introduction

Acupuncture is a diagnostic and therapeutic method rooted in traditional Chinese medicine. Numerous high-quality randomized controlled trials (RCTs) and clinical practice guidelines have demonstrated the clear clinical efficacy of acupuncture[1–5], but its mechanism of action is complex and cannot be adequately explained by a single mechanism. Overall, the effects of acupuncture can be divided into three stages: “sensation-transmission-effect,” namely, activation of the acupoint area (sensation) — transmission and integration along complex network pathways (transmission) — regulation of the target organ (effect)[6]. Acupoint is the initial response site to the acupuncture stimulation. The activation of the acupoint area is the starting point of this entire three-stage process of acupuncture effect. It is crucial to reveal the mechanism of action of acupuncture from the initial site.

Acupuncture can produce a sensation of sourness, numbness, heaviness, and distension, known as “deqi,” which is considered a key factor in the therapeutic effects of acupuncture. Jiang Zhenyu et al. found that when subjects experienced the deqi sensation after acupuncture at the LI4 and LI10 acupoints, significant analgesic effects were observed. Other studies have shown that the deqi sensation from acupuncture can significantly reduce the average number of headache days in patients with chronic tension-type headaches[7]. Acupuncture at the SP6 point with the deqi sensation can significantly reduce menstrual pain in patients with primary dysmenorrhea due to cold and dampness stagnation, while the acupuncture group without the deqi sensation showed no significant therapeutic effect[8]; Sun R et al. found that compared with the acupuncture group without deqi, the acupuncture group with deqi significantly improved the symptoms of patients with functional dyspepsia[9]. The above studies all show that the acupuncture effect is better when deqi is obtained than when it is not obtained. Deqi is a subjective sensation, but it is also accompanied by some objective reactions, such as local muscle electrical discharge at the acupoint when deqi is obtained[10], and an increase in the pulling force of the needle[11]. Muscle electrical discharge in the acupoint area when deqi is obtained indicates local muscle contraction induced by acupuncture. When muscles contract, the tightness between muscle fibers and the needle increases, leading to an increase in friction between the muscle and the needle. When the needle is pulled upward, the pulling force of the needle also increases accordingly. Thus, the objective reactions during deqi are all related to muscle contraction. Lu Fengyan's research also showed that the primary sensations associated with deqi, such as distension, dull pain, and acidity, as well as the practitioner's sensation of heaviness in the hand, primarily originate from muscle tissue. The participant's sensation of distension often occurs concurrently with the practitioner's sensation of heaviness in the hand[10], suggesting that muscle contraction induced by acupuncture may be related to deqi and acupuncture effects.

Transient receptor potential vanilloid 1 (TRPV1). was first identified as a temperature-sensitive ion channel that can be activated by various physical and chemical stimuli, such as heat (T > 43 °C), protons (pH < 5.9), oxytocin, and various inflammatory mediators[12]. Subsequent studies by other researchers found that mechanical stimuli such as stretching can also activate this channel[13]. Since TRPV1 is an ion channel located on the cell membrane, mechanical stimuli can alter the tension of the lipid bilayer, thereby changing the tension of the cell membrane and affecting the opening of the TRPV1 channel, indicating that TRPV1 is a multisensory ion channel. Research has shown that TRPV1 is primarily distributed at the nerve endings of Aδ and C fibers in primary sensory neurons that detect noxious signals[14], and is highly expressed in type IV afferent fibers that innervate skeletal muscle[15]. Acupuncture, as a mechanical physical stimulus, can induce the local release of numerous chemical stimuli[16], such as neuropeptide P[17], substance P (prostaglandin)[18], ATP[19], and IL-1β[20]. These chemical stimuli activate TRPV1 channels, thereby further propagating the acupuncture-induced stimulus signal and influencing the therapeutic acupuncture effect. Some studies have demonstrated that knocking out TRPV1 in Complete Freund’s Adjuvant (CFA)-induced mice diminishes the analgesic effect of acupuncture on mice[21]. Conversely, other studies have found that injecting TRPV1 agonists into acupoint areas can mimic the analgesic effect of acupuncture[22, 23]. These studies indicate that TRPV1 in acupoint areas mediates the analgesic effect of acupuncture. However, whether the local role of TRPV1 in acupoints is universal remains unclear, investigating this question will help elucidate the common features underling the mechanism of acupuncture therapy.

Coronary heart disease refers to a type of heart disease caused by coronary artery atherosclerosis, which leads to vascular narrowing or blockage and consequently resulting in myocardial ischemia, hypoxia, or necrosis[24]. Specifically, acute myocardial infarction (AMI) is a severe form of this disease, characterized by myocardial tissue ischemia and necrosis due to acute obstruction of the coronary artery [25]. Patients typically present with chest pain, often accompanied by shortness of breath, nausea, and sweating. Moreover, AMI can lead to a series of severe complications, such as heart failure, cardiogenic shock and malignant arrhythmias, making it one of the most common life-threatening cardiovascular diseases in clinical practice[26]. Acupuncture shows potential in the treatment of AMI. For example, acupuncture can significantly reduce the frequency of episodes in patients with severe angina pectoris[27], and acupuncture at PC6 (Neiguan) for 15 min can significantly increase the left ventricular ejection fraction(LVEF) in patients with coronary heart disease[28]; continuous electroacupuncture pretreatment(EAP) for 5 days prior to heart valve replacement surgery can reduce the severity of myocardial ischemia–reperfusion injury (MIRI)[29]; acupuncture can reduce the incidence of myocardial infarction after percutaneous coronary intervention[30]. The present study will utilize the platform of acupuncture at PC6 to improve cardiac function in AMI rats to explore the role of TRPV1 in the muscle layer of PC6 during acupuncture. This will provide evidence from another disease model for TRPV1-mediated effects in acupoint areas, while also offering evidence to demonstrate its universal role. We hypothesize that acupuncture into the muscle layer exerts better therapeutic effects than acupuncture into the subcutaneous layer, based on evidence that the sensation of “deqi” is generated in the muscle layer. TRPV1 in the muscle layer is more activated after muscle contraction induced by acupuncture, thereby amplifying the transmission of acupuncture signals and producing an improvement in cardiac function in AMI rats.

## Materials and methods

### Experimental animals

Male SD rats, aged 6–7 weeks and weighing 190 ± 20 g, were supplied by Beijing Vital River Laboratory Animal Technology Co., Ltd., [License No.: SYXK (Tianjin) 2020–0005]. The animals were housed in the Animal Care Center of the Experimental Animal Center under controlled conditions: temperature was maintained at 25 ± 2 °C, relative humidity at 50–60%, and a 12-h light/dark cycle. All rats were acclimatized individually for one week prior to experimentation. Standard laboratory chow and water were provided ad libitum under hygienic conditions. This study was conducted in strict accordance with the Guide for the Care and Use of Laboratory Animals and was approved by the Animal Ethics Committee of Tianjin University of Traditional Chinese Medicine (Ethical Approval No.: TCM-LAEC2019062).

### Experimental design

Experiment 1: Since acupuncture at the muscle layer is more likely to induce the deqi sensation compared to acupuncture at the subcutaneous tissue layer, we first aim to investigate whether the acupuncture effect corresponds to this difference. Sprague–Dawley (SD) rats were used to establish an AMI model and randomly divided into four groups (n = 6–7 per group): (1) Sham group [sham operation performed, no acupuncture] (2) AMI group [AMI modeling performed, no acupuncture], (3) AMI + ACU-M group [AMI modeling performed, acupuncture at the muscle layer of PC6], (4) AMI + ACU-S group [AMI modeling performed, acupuncture at the subcutaneous layer of PC6]. After 7 days of intervention, cardiac function changes were observed using a small animal ultrasound imaging system, myocardial ischemia area was detected via 2,3,5-triphenyltetrazolium chloride (TTC) staining, and norepinephrine (NE) content was measured using an enzyme linked immunosorbent assay (ELISA) kit. These assessments aimed to compare the therapeutic efficacy of the two acupuncture interventions.

Experiment 2: To explore whether the normal muscle contraction function influences the acupuncture effect, muscle relaxants were locally administered at acupoints to induce loss of normal muscle contractility, with subsequent observation of the resultant impact on the acupuncture effect and simultaneous detection of changes in TRPV1 at the local acupoint area. SD rats were randomly divided into four groups (n = 6–7 per group): (1) Sham + Vehicle group [sham operation performed, injected with 0.9% NaCl at PC6, no acupuncture], (2) AMI + Vehicle group [AMI modeling performed, injected with 0.9% NaCl at PC6, no acupuncture], (3) ACU + Vehicle group [AMI modeling performed, injected with 0.9% NaCl at PC6, acupuncture performed], (4) ACU + Scc group [AMI modeling performed, injected with succinylcholine chloride (Scc, muscle relaxant, 0.75 mg/ml) at PC6, acupuncture performed at PC6]. After sham operation and successful establishment of AMI modeling, rats in each group were injected with 20 μl of the corresponding solution at PC6. Acupuncture was performed at PC6 10 min after injection in groups (3) and (4). After 7 days of intervention, cardiac function was assessed and myocardial ischemia area were measured in all groups. Additionally, tissue from the PC6 acupoint area and C5-T1 segment dorsal root ganglia (DRG) was collected from each group. Immunofluorescence (IF) and Western blotting (WB) were used to detect changes in TRPV1 in the PC6 acupoint area and C5-T1 DRG.

Experiment 3: This experiment was designed to evaluate whether TRPV1 in the acupoint area mediates the effect of acupuncture at PC6 on improving cardiac function in AMI rats. SD rats were divided into three groups (n = 10 per group): (1) AMI + DMSO group [AMI modeling performed, injected with dimethyl sulfoxide (DMSO, TRPV1 inhibitor solvent) at PC6, no acupuncture], (2) AMI + DMSO + ACU group [AMI modeling performed, injected with DMSO at PC6, followed by acupuncture at PC6], (3) AMI + CPZ + ACU group [AMI modeling performed, injected with capsazepine (CPZ, TRPV1 inhibitor, 0.5 mg/ml) at PC6, followed by acupuncture at PC6]. After sham operation and successful establishment of AMI modeling, rats in each group were injected with 20 μl of the corresponding solution at PC6. Acupuncture was performed at PC6 10 min after injection. After 7 days of intervention, the same indicators as in Experiment 2 were measured, including cardiac function, myocardial ischemic area, TRPV1 expression in the PC6 acupoint area and C5-T1 DRG.

### Animal model

Prior to surgery, rats were fasted and water-deprived for 8 h. They were placed in an anesthesia induction box, and the concentration of the anesthetic agent isoflurane in the gas anesthesia machine was adjusted to 3–4%, with oxygen concentration set to approximately 2%. Once the rats were fully anesthetized, the animal was secured in a supine position on the surgical table and fitted with an anesthesia mask. The isoflurane concentration was then maintained at 1.5%–2%. The thoracic area was shaved and disinfected with iodine, centered approximately 0.5 cm left of the midline where the heartbeat was strongest. A transverse incision approximately 1 cm in length was made at this location. The pectoralis major and minor muscles were bluntly dissected to expose the rib cage. A curved vascular clamp was gently inserted between the fourth and fifth ribs until a loss of resistance was felt, and the intercostal space was carefully widened to visualize the beating heart. The heart was rapidly exteriorized to expose the left atrial appendage and pulmonary conus. The ligation point was identified at one-third of the distance from the base to the apex along the line between the left atrial appendage and the pulmonary artery. A 5–0 suture needle was used to ligate the myocardium at a depth of approximately 0.1 cm and a width of 0.2 cm. A standard lead II electrocardiogram (ECG) was used to monitor cardiac activity throughout the experiments. Successful modeling was confirmed by the observation of whitening of the ligated segments of the heart, accompanied by weakened pulses and a significant upward arching of the ST segment on the ECG[31]. Then the heart was promptly returned to the thoracic cavity. The anesthesia mask was removed, and once spontaneous heartbeat and respiration resumed, the muscle and skin layers were sutured.

### Acupuncture intervention

To enable acupuncture administration in awake rats, a specialized rat restraint vest was designed to improve immobilization. The acupoint targeted in this study was PC6, situated between the tendons of the palmaris longus and the flexor carpi radialis, approximately two cun proximal to the distal wrist crease on the palmar side[32]. The underlying musculature at the PC6 site in rats includes the extensor carpi radialis, extensor carpi ulnaris, and extensor digitorum communis. Following the establishment of the AMI model, the rats were restrained, and the bilateral PC6 acupoints were exposed and disinfected with iodine. A filiform needle (0.25 × 13 mm; Beijing Zhongyan Taihe Medical Devices Co., Ltd.) was used for acupuncture. In the AMI + ACU-M group, the needle was inserted to a depth of approximately 0.4 cm. After the “deqi” sensation was achieved—characterized by a feeling of tenseness around the needle from the practitioner[33], the needle was manipulated with a twisting technique. The amplitude of each twisting motion was 360°, specifically involving a 360° clockwise twist followed by a 360° counterclockwise twist. One such bidirectional movement was designated as one twisting cycle, and the twisting manipulation was performed at a frequency of 120 cycles per minute. The needle was retained for 8 min after 2 min of manipulation; this process was repeated once, resulting in a total needle retention time of 20 min. The treatment was administered once daily for 7 consecutive days. For the AMI + ACU-S group, the needle was inserted subcutaneously without deeper manipulation; all other procedures matched those of the AMI + ACU-M group. Rats in the Sham and AMI groups received no acupuncture treatment. To control for non-treatment factors, these animals were also restrained using custom-made vests for 20 min, consistent with the handling of the acupuncture groups.

### Electrocardiogram recording

After anesthetizing the rat, place the animal in a supine position. Set up a PowerLab physiological signal acquisition system to monitor the electrocardiogram (ECG). Connect the electrodes in a standard limb lead II configuration: attach the positive electrode to the left hind ankle, the negative electrode to the right forelimb wrist, and the ground electrode to the right hind ankle. ECG recordings should be taken both 24 h prior to and immediately following the modeling procedure. An ST-segment elevation ≥ 0.2 mV was considered indicative of successful AMI modeling.

### Acupoint injection of drugs

The muscle relaxant used in Experiment 2 was Scc, a fast-acting depolarizing neuromuscular blocking agent. It acts specifically on the postsynaptic membrane of the neuromuscular junction by targeting nicotinic acetylcholine receptors, resulting in a depolarizing block. Scc is commonly employed in clinical settings such as surgery, bronchoscopy, and esophagoscopy. However, high-dose subcutaneous administration in rats can induce histopathological damage in organs including the heart, kidneys, and lungs [34]. Based on prior toxicity studies, the median lethal dose (LD50) in rats is 1.59 mg/kg [35], Scc powder was dissolved in 0.9% NaCl to achieve a concentration of 0.75 mg/ml, and a volume of 20 μl was injected per site. For Experiment 3, the TRPV1 receptor channel blocker selected was CPZ, a competitive capsaicin antagonist and synthetic analog of excitotoxic compounds affecting sensory neurons. The dosage was converted from previous mouse studies to an equivalent intramuscular dose for rats^36^. The final injection concentration was 0.5 mg/ml, administered at 20 μl per site.

### Echocardiography analysis

Cardiac function in rats was assessed using a small-animal ultrasound imaging system. The rats were placed under anesthesia and positioned supine on the imaging platform. The chest was shaved and applied with ultrasound coupling gel. Using the ultrasound interface, B-mode was selected initially. The probe was positioned over the apical beat region and adjusted until both the cardiac apex and the left ventricular outflow tract were clearly visualized. The imaging mode was then switched to M-mode, and the reference lines were aligned to optimize visualization of the left ventricular anterior and posterior walls. The reference lines were positioned between the chordae tendineae and papillary muscles, after which the images were saved by selecting “SAVE CLIP”. This echocardiographic examination allowed for the assessment of changes in left ventricular end-diastolic and end-systolic volumes, as well as calculation of the LVEF and left ventricular fractional shortening (LVFS).

### TTC staining

Seven days after the intervention, the rats were euthanized, and their hearts were promptly excised and placed in pre-labeled culture dishes in a standardized orientation. The dishes were then transferred to a –20 °C freezer and frozen for 30 min to fix the cardiac structure. Subsequently, the samples were stored in a –80 °C freezer until further processing. For analysis, the heart tissues were retrieved from the –80 °C freezer and sectioned uniformly from the apex to the ligation site into five slices, each approximately 0.2 cm thick. The slices were immersed in TTC staining solution at 37 °C for 20 min and gently agitated every 5 min. After staining, the slices were removed and fixed overnight. The fixed heart slices were then arranged in sequence from apex to base on black cardstock, maintaining uniform spacing and consistent orientation. Photographs were taken under adequate lighting using a smartphone and saved for analysis. The ischemic area of the heart was assessed using Image-Pro Plus analysis software. The percentage of ischemic area was calculated as follows: (ischemic area of each slice/total myocardial area of the same slice) × 100%.

### Serum NE detection

Follow the instructions in the ELISA kit NE (Quanzhou Ruixin Biotechnology Co., Ltd.) for specific operations.

### Immunofluorescence staining

IF of Acupoint Tissue: Seven days post-intervention, the rats were anesthetized and perfused successfully with 4% paraformaldehyde. Using a sterile scalpel, a tissue block measuring 0.5 cm × 0.5 cm × 0.5 cm was excised from the PC6 acupoint area, including the skin, subcutaneous tissue, and muscle layers. The samples were placed in 4% tissue fixative and stored at 4 °C. DRG Tissue Sampling: Similarly, seven days after the intervention, the rats were anesthetized and perfused with 4% paraformaldehyde. The spinal segment from C5 to T1 was identified, and the corresponding DRG tissues were collected. These were placed in 4% formalin solution and stored at 4 °C until embedding. Both the acupoint and DRG tissues were embedded in paraffin, sectioned at a thickness of 0.7 cm, dried, dewaxed, and subjected to antigen retrieval. Autofluorescence quencher solution A was applied for 30 min, followed by three 5-min washes with distilled water. The sections were then blocked with 5% BSA for 30 min. Primary antibodies—anti-TRPV1 (1:400) and anti-c-FOS (1:600)—were applied and incubated overnight at 4 °C. The following day, the sections were warmed to room temperature for 30 min and washed five times with PBS for 5 min each. Secondary antibodies—goat anti-rabbit IgG H&L (Alexa Fluor® 488, 1:400) and goat anti-mouse IgG H&L (Alexa Fluor® 594, 1:400)—were applied in the dark for 50 min, followed by another five 5-min PBS washes. Finally, DAPI staining was performed for 8 min, and the sections were washed five times with PBS (5 min each). An appropriate amount of anti-fade mounting medium was applied, and coverslips were placed. Images were acquired using a Leica fluorescence microscope. ImageJ software was used for analysis: the positive expression area of TRPV1 in the acupoint region was quantified, and the percentage of DRG neurons co-expressing TRPV1 and c-FOS was calculated as (number of TRPV1⁺ and c-FOS⁺ neurons)/(number of TRPV1⁺ neurons) × 100%. All statistical analyses were performed using the ImageJ counting tool.

### Western blot

WB analysis of acupoint tissue: Seven days after the intervention, the rats were euthanized. A tissue block measuring 0.5 cm × 0.5 cm × 0.5 cm was excised from the PC6 acupoint area using a sterile scalpel, comprising skin, subcutaneous tissue, and muscle layers. The samples were stored at − 80 °C for subsequent analysis. For protein extraction, the acupoint tissue samples were homogenized in protein lysis buffer. The total protein concentration was determined using a BCA assay. Subsequently, 10 μL of each protein sample was mixed with 5 μL of protein ladder and subjected to electrophoresis. Following separation, proteins were transferred to a membrane, which was then blocked with rapid blocking buffer for 10 min and washed. The membrane was incubated with primary antibody overnight at 4 °C, followed by three washes with TBST. A secondary antibody was applied and incubated for 1 h at room temperature, after which the membrane was washed three times with TBST. Protein bands were visualized using ECL chemiluminescent substrate and exposed for imaging. The gray values of the target bands were quantified using ImageJ software.

### Statistics

Data were analyzed using SPSS 26.0 statistical software. First, For within-group paired comparisons (e.g., Pre-AMI vs. AMI in the same cohort of rats), a Paired t-test was used if the data were normally distributed; otherwise, a Wilcoxon signed-rank test was applied.normality tests were performed for each outcome variable in each experimental group. For between-group comparisons (e.g., comparisons among Sham, AMI, and acupuncture intervention groups), after confirming that the data met the criteria for normal distribution, one-way analysis of variance (ANOVA) was used. Homogeneity of variance tests were conducted; if the variances were homogeneous, the LSD test was used for post-hoc comparisons; if the variances were heterogeneous, Dunnett’s T3 test was used. When the data did not meet the criteria for normal distribution, nonparametric Kruskal–Wallis tests were performed. A *P*-value of < 0.05 was considered statistically significant, and a *P*-value of < 0.01 was considered highly statistically significant.

## Results

### Acupuncture at the muscle layer of PC6 improves cardiac function in AMI rats more effectively than subcutaneous layer acupuncture

**Fig.1:**
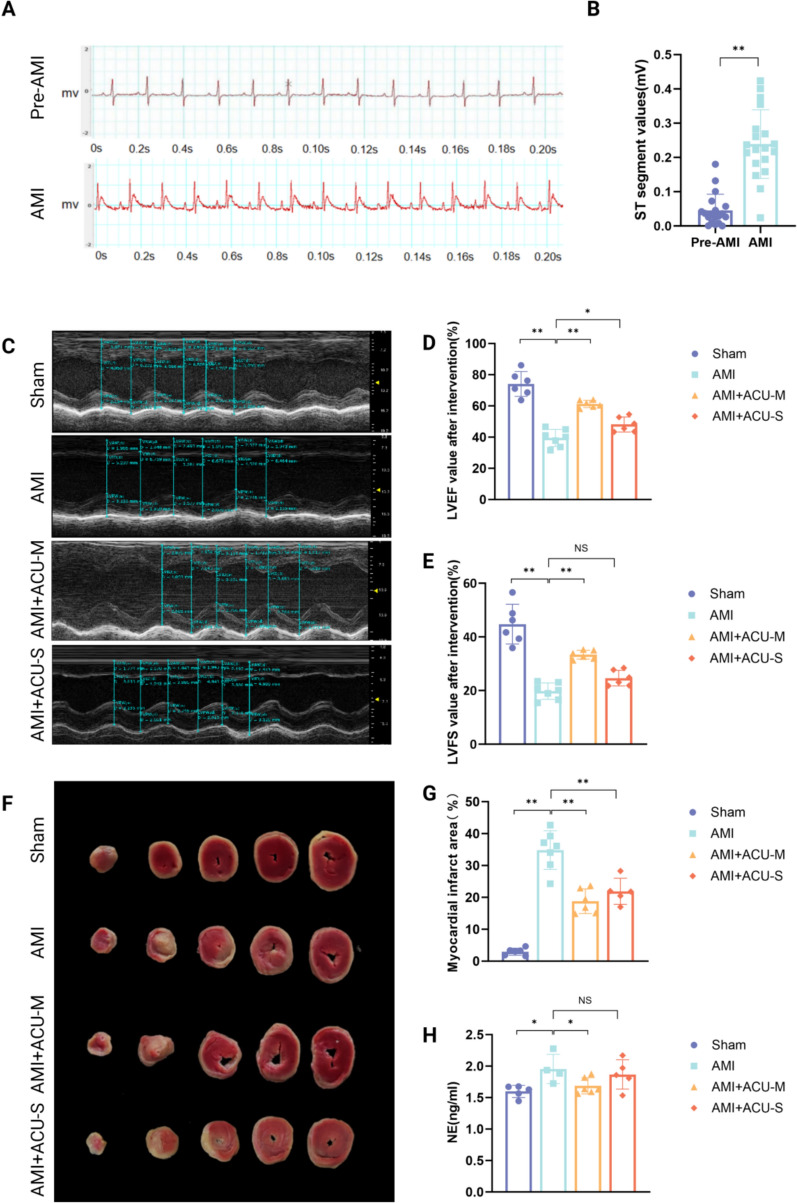
Superior effects of muscle-layer acupuncture at PC6 versus subcutaneous acupuncture on cardiac function in AMI rats. **A** Representative ECG of rats before and after AMI.. **B** Analysis of ECG data before and after AMI in rats (n = 19, paired analysis). **C** Representative echocardiography images of rat hearts.. **D** Comparison of LVEF values among groups (n = 6–7 per group). **E** Comparison of LVFS values among groups (n = 6–7 per group). **F** Representative images of myocardial ischemia area in rats. **G** Effects of different intervention methods on myocardial ischemia area in rats (n = 6–7 per group). **H** Effects of different intervention methods on rat serum NE levels (n = 5–7 per group). *P < 0.05, **P < 0.01

To confirm the success of AMI model induction, electrocardiogram (ECG) signals of rats were collected using a PowerLab physiological recording system and subjected to subsequent quantitative analysis. The ECG (Fig. 1A-B) recordings of AMI rats immediately after successful modeling revealed a marked elevation in the ST-segment amplitude compared with the baseline ECG signals obtained before AMI induction. Statistical analysis verified that this elevation of the ST-segment was significant, which met the standard for successful AMI model establishment and thus validated the reliability of the model used in this study.

According to echocardiography (Fig. 1C), after 7 days of intervention, rats in the Sham group exhibited a thicker left ventricular anterior wall with normal contractile and diastolic function. Compared with the Sham group, the AMI group showed significant thinning of the left ventricular anterior wall and weakened contraction, accompanied by impaired contractile and diastolic function. Compared with the AMI group, the AMI + ACU-M group displayed a significant increase in left ventricular anterior wall thickness and marked recovery of ventricular function. By contrast, the AMI + ACU-S group showed slight recovery of left ventricular contraction and diastolic function, but to a lesser extent than the AMI + ACU-M group.

According to the results of echocardiography (Fig. 1D-E), TTC staining (Fig. 1F-G), and ELISA assay kit (NE) detection of serum NE levels (Fig. 1H), after 7 days of intervention, compared with the Sham group, the LVEF values of the AMI group were significantly reduced, LVFS values were significantly reduced, myocardial ischemia area was significantly increased, and serum NE levels were elevated; Compared with the AMI group, the AMI + ACU-M group showed significantly increased LVEF values, significantly increased LVFS values, significantly reduced myocardial ischemia area, and decreased serum NE levels; Compared with the AMI group, the AMI + ACU-S group had increased LVEF values, no significant changes in LVFS values, significantly reduced myocardial ischemia area, and no significant changes in serum NE levels; Acupuncture at PC6 improved rat cardiac function and myocardial infarction area, with the AMI + ACU-M showing a more pronounced improvement trend. Muscle layer acupuncture reduced serum NE levels in AMI rats and inhibited sympathetic nervous system excitability, whereas subcutaneous acupuncture did not exhibit these effects. This indicates th394at the key site for acupuncture effects is the muscle layer.

## Acupoint injection of Scc reverses the improvement of cardiac function and TRPV1 expression in the acupoint area of AMI rats

**Fig. 2:**
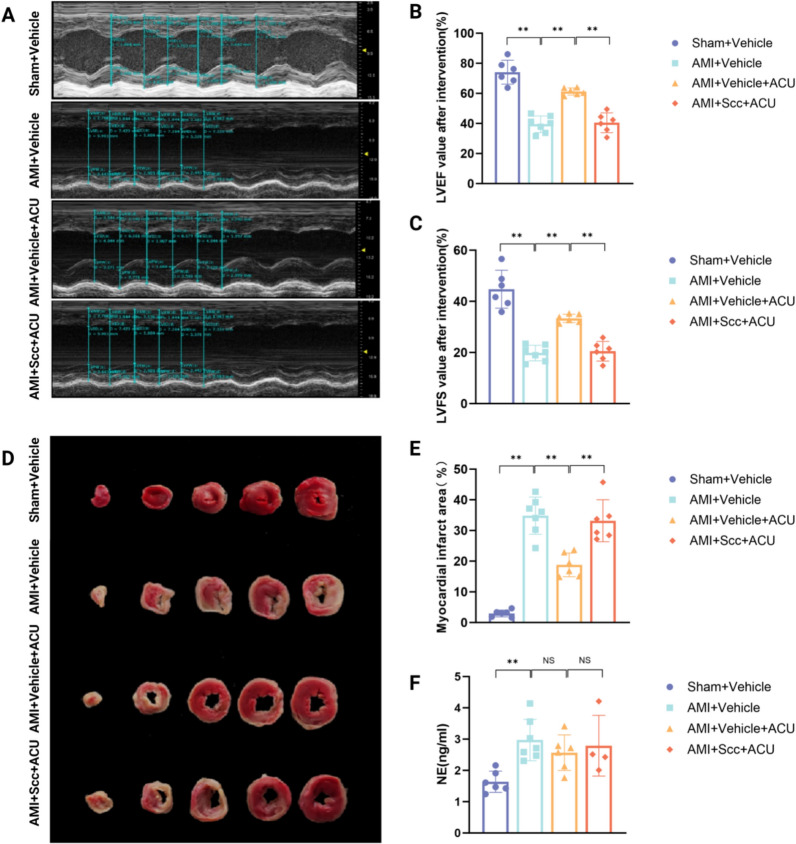
Scc abolishes the cardioprotective effect of acupuncture in AMI rats. **A** Representative echocardiography images of rat hearts. **B** Effect of PC6 injection of Scc on LVEF values in rats with AMI undergoing acupuncture intervention (n = 6–7 per group). **C** Effect of PC6 injection of Scc on LVFS values in rats with AMI undergoing acupuncture intervention (n = 6–7 per group). **D** Representative images of myocardial ischemia area in rats. **E** Effect of PC6 injection of Scc on myocardial ischemia area in rats with AMI undergoing acupuncture intervention (n = 6–7 per group). **F** Effect of PC6 injection of Scc on serum NE levels in acupuncture-treated AMI rats (n = 4–7 per group). * *P* < 0.05, ** *P* < 0.01

Acupoint injection of Scc had no effect on cardiac function, myocardial ischemia area, etc., in both normal rats and AMI rats (see Additional file 1). Based on this finding, echocardiography (Fig. 2A) showed that after 7 days of intervention, the left ventricular anterior wall thickness in the Sham + Vehicle group was thicker and exhibited normal contractile and diastolic function. Compared with the Sham + Vehicle group, the left ventricular anterior wall of the AMI + Vehicle group was significantly thinner, with impaired contractile and diastolic function. Compared with the AMI + Vehicle group, the AMI + Vehicle + ACU group showed a significant increase in left ventricular anterior wall thickness and marked recovery of ventricular contractile and diastolic function. Compared with the AMI + Vehicle + ACU group, the left ventricular anterior wall in the AMI + Scc + ACU group was significantly thinner, with notably weaker contractile and diastolic functions.

According to the results of echocardiography (Fig. 2B-C), TTC staining (Fig. 2D-E), and ELISA assay kit (NE) detection of serum NE levels (Fig. 2F), after 7 days of intervention: compared with the Sham + Vehicle group, the AMI + Vehicle group showed significantly reduced LVEF and LVFS values, a significantly increased percentage of myocardial ischemia area, and elevated serum NE levels; compared with the AMI + Vehicle group, the AMI + Vehicle + ACU group exhibited significantly increased LVEF and LVFS values, a significantly decreased percentage of myocardial ischemia area, and a trend toward decreased serum NE levels (without statistical significance); compared with the AMI + Vehicle + ACU, the AMI + Scc + ACU group displated significantly decreased LVEF and LVFS values, a significant increased percentage of myocardial ischemia area, and a trend toward increased serum NE levels (without statistical significance). Taken together, these results indicate that acupoint injection of Scc at PC6 can reverse the beneficial effects of acupuncture on cardiac function and myocardial ischemia area in AMI rats, and trends to reverse the inhibitory effect of acupuncture on sympathetic nerve excitability in these rats.

**Fig. 3:**
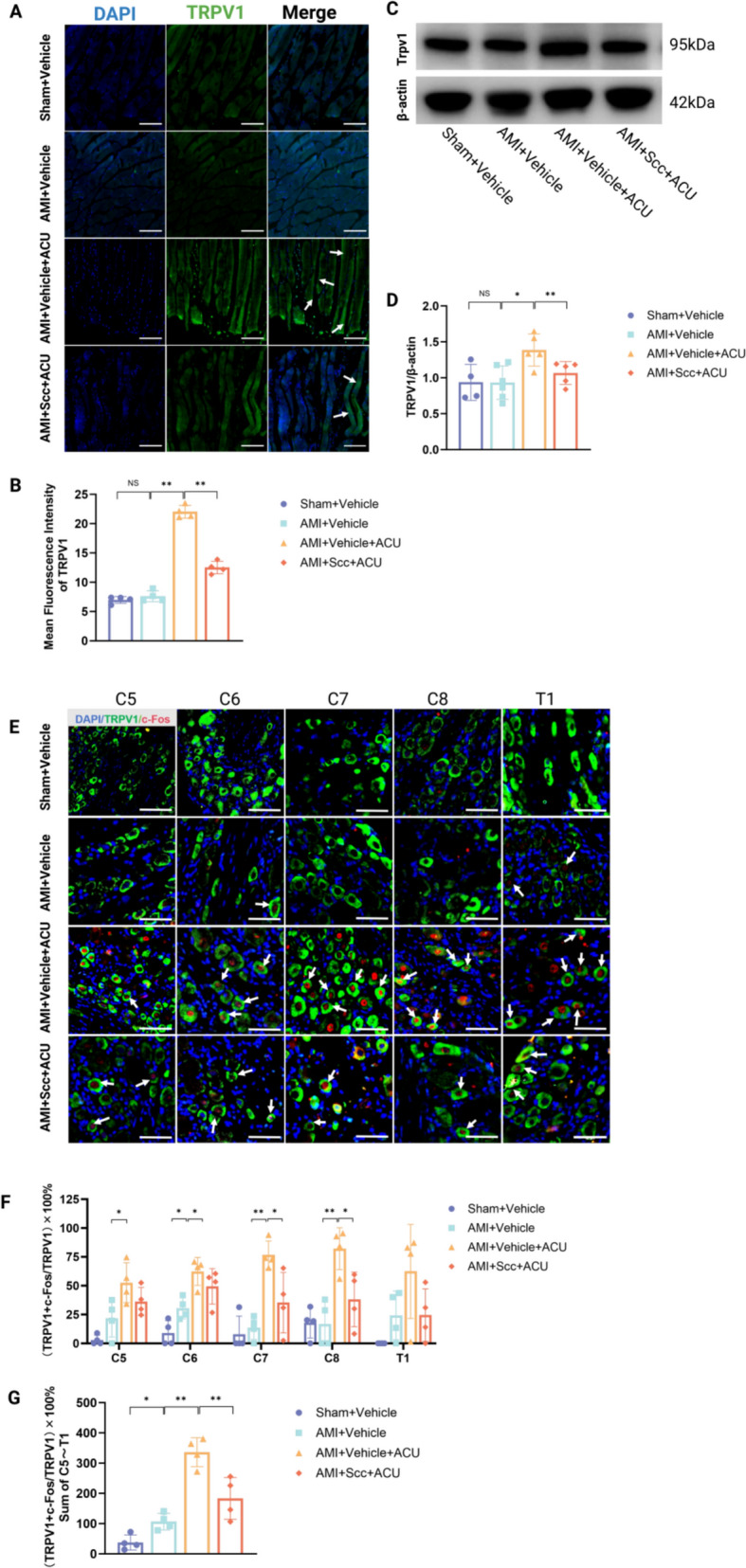
Scc inhibits TRPV1 upregulation in acupoint and DRG of AMI rats. **A** Representative immunofluorescence images of TRPV1-positive expression in PC6 (Scale bars: 100um. Magnification: 10 ×). White arrows indicate TRPV1-positive expression in the muscle layer. **B** Effect of PC6 injection of Scc on the area of TRPV1 positive expression in the acupoint area of AMI rats treated with acupuncture (n = 4 per group) **C** Representative Western blot of TRPV1 protein expression in PC6. **D** Effect of PC6 injection of muscle relaxant on TRPV1 protein expression in the acupoint area of AMI rats treated with acupuncture (n = 4–6 per group) **E** Representative immunofluorescence co-staining images of TRPV1 and c-Fos in the C5–T1 segment of the DRG (Scale bars: 50um. Magnification: 20 ×). White arrows indicate co-labeling of TRPV1 and c-Fos.. **F** Effect of PC6 injection of Scc on TRPV1 neurons in the C5-T1 segment DRG of AMI rats following acupuncture intervention (n = 4 per group) **G** Effect of injection of Scc into the acupoint area on TRPV1 neurons in the DRG of AMI rats treated with acupuncture (n = 4 per group). *P < 0.05, ** *P* < 0.01

After acupoint injection of Scc and 7 days of intervention, IF staining of the PC6 area (Fig. 3A, B), WB analysis (Fig. 3C, D), IF staining of the C5-T1 segment DRG (Fig. 3E–G) showed the following results: Compared with the Sham + Vehicle group, the AMI + Vehicle group exhibited no significant changes in the TRPV1 protein expression in the muscle layer of the acupoint area, as determined by both IF staining and WB. In the DRG, the AMI + Vehicle group showed increased activation of TRPV1-positive neurons in the C6 segment, with no significant changes in the C5 or C7-T1 segments, and no significant difference in the total percentage of TRPV1-positive cells across the DRG. Compared with the AMI + Vehicle group, the AMI + Vehicle + ACU group displayed a significant increase in TRPV1 protein expression in the muscle layer of acupoint area. In the DRG, this group showed significantly increased activation of TRPV1-positive neurons in the C5-C8 segments and a significant increase in the total percentage of TRPV1-positive cells. Compared with the AMI + Vehicle + ACU group, the AMI + Scc + ACU group showed a significant decrease in TRPV1 protein expression in the muscle layer of the acupoint area and a significant reduction in TRPV1 protein expression in the muscle layer of the acupoint area. In the DRG, the percentage of TRPV1-positive cells in the C7 and C8 segments was significantly reduced, along with a reduction in the total percentage of TRPV1-positive cells. Collectively, these findings indicate that acupoint injection of a muscle relaxant at PC6 can reverse the upregulation of TRPV1 in the muscle layer of the acupoint area and the activation of TRPV1 in the DRG of AMI rats.

## Injection of CPZ into the PC6 muscle layer antagonizes acupuncture-induced improvement of cardiac function in AMI rats

**Fig. 4:**
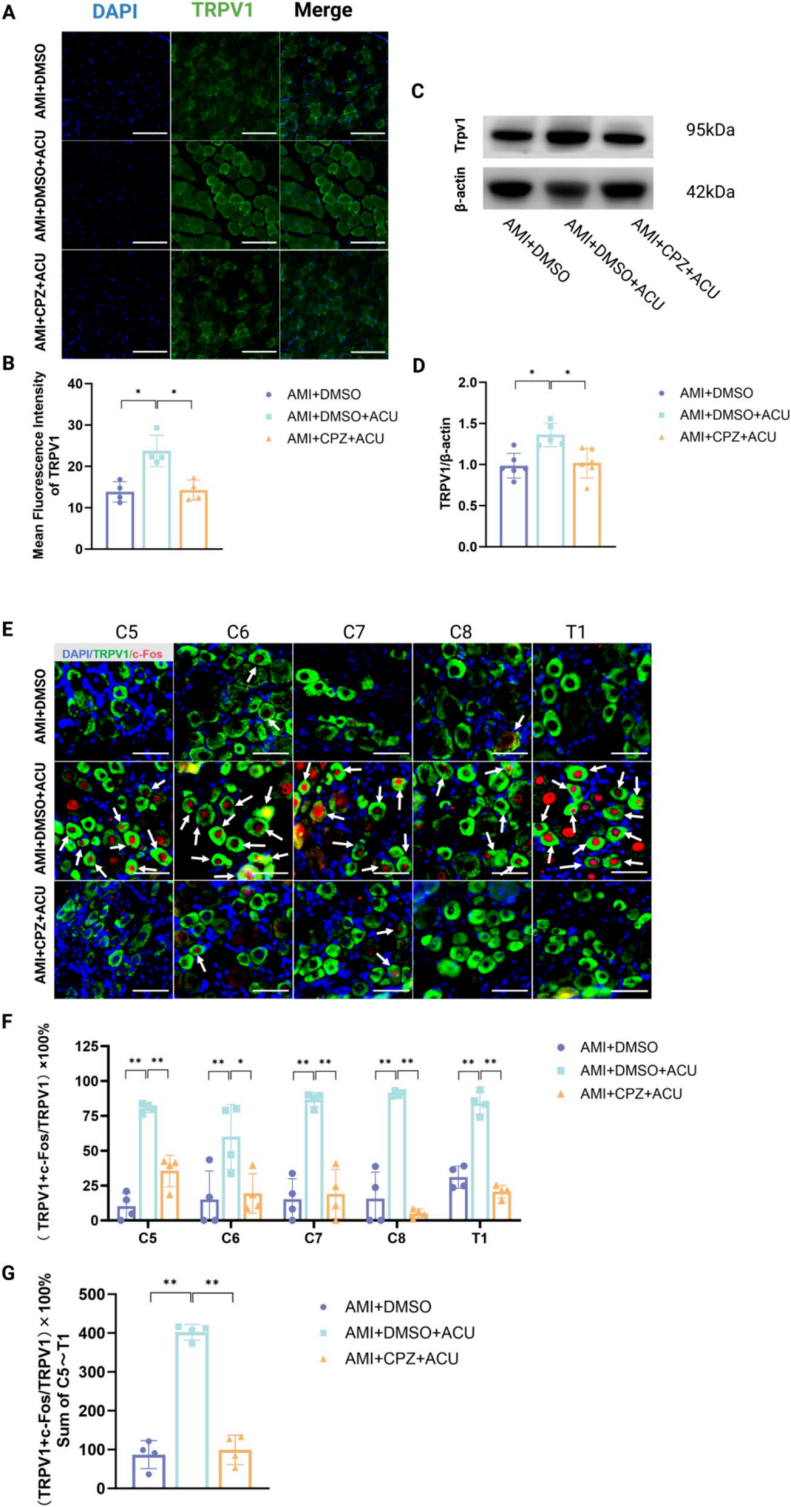
CPZ inhibits acupuncture-induced TRPV1 upregulation in acupoint and DRG. **A** Representative immunofluorescence images of TRPV1-positive expression in PC6 (Scale bars: 100um. Magnification: 10 ×). **B** Effect of CPZ injection into PC6 on the area of TRPV1 positive expression in the acupoint area of AMI rats treated with acupuncture (n = 4 per group) **C** Representative Western blot of TRPV1 protein expression in PC6. **D** Effect of CPZ injection into PC6 on the area of TRPV1 positive expression in the acupoint area of AMI rats treated with acupuncture (n = 5–6 per group) **E** Representative immunofluorescence co-staining images of TRPV1 (green) and c-Fos (red) in the C5–T1 segment of the DRG (Scale bars: 50um. Magnification: 20 ×). White arrows indicate co-labeling of TRPV1 and c-Fos. **F** Effect of CPZ injection into PC6 on TRPV1 neurons in the C5-T1 segment DRG of AMI rats treated with acupuncture (n = 4 per group). **G** Effect of CPZ injection into the acupoint area on TRPV1 neurons in the DRG of AMI rats treated with acupuncture (n = 4 per group). * *P* < 0.05, ** *P* < 0.01

After 7 days of intervention with CPZ injection into the acupoint area, compared with the AMI + DMSO group, AMI + DMSO + ACU group showed a significant increase in TRPV1 protein expression in the muscle layer of acupoint area, as determined both by IF staining (Fig. 4A-B) and WB (Fig. 4C-D), along with a significant increase in the proportion of activated TRPV1 neurons and their total number in the C5-T1 segment DRG (Fig. 4E-G). In contrast, compared with the AMI + DMSO + ACU group, the AMI + CPZ + ACU group exhibited a significant decrease in TRPV1 protein expression in the acupoint area both with IF (Fig. 4A-B) staining and WB (Fig. 4C, D). In the C5-T1 DRG, the proportion of TRPV1-positive neurons was significantly reduced (Fig. 4E–F), and a significant reduction was also observed in the total number of activated TRPV1 neurons (Fig. 4G). These results indicate that injection of CPZ at PC6 can reverse the upregulation of TRPV1 expression in the acupoint area and the activation of TRPV1 neurons in the DRG of AMI rats.

**Fig. 5:**
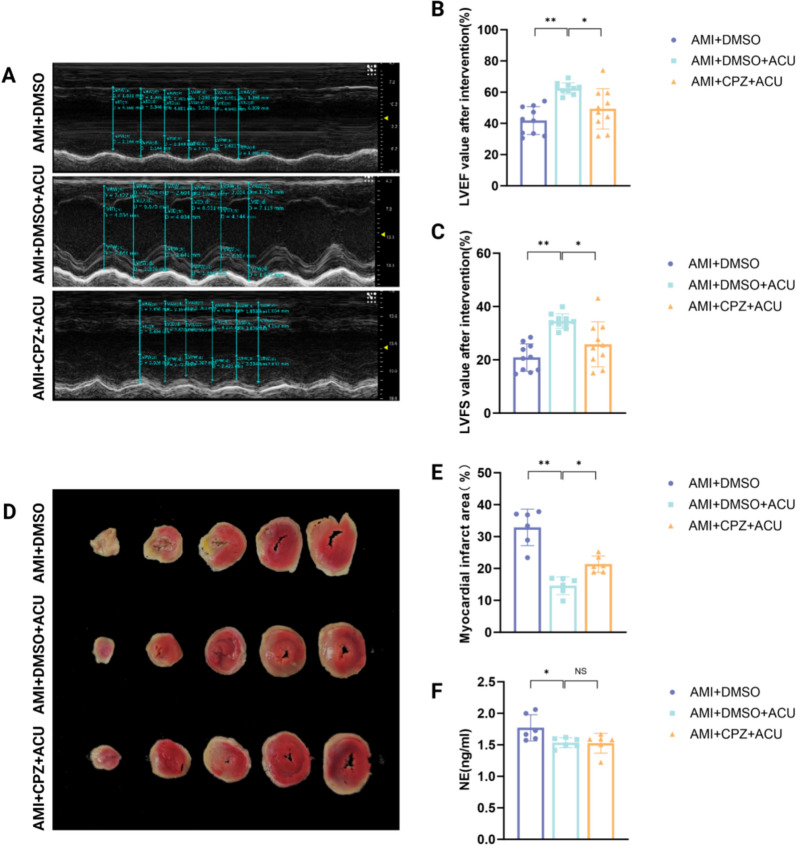
CPZ partially reverses acupuncture-induced cardioprotection in AMI rats. **A** Representative echocardiography images of rat hearts.** B** Effect of CPZ injection at PC6 on LVEF values in acupuncture-treated AMI rats (n = 10 per group). **C** Effect of CPZ injection at PC6 on LVFS values in acupuncture-treated AMI rats (n = 10 per group). **D** Representative images of myocardial ischemia area in rats. **E** Effect of CPZ injection at PC6 on myocardial ischemia area in acupuncture-treated AMI rats (n = 6 per group). **F** Effect of CPZ injection at PC6 on serum NE levels in acupuncture-treated AMI rats (n = 6 per group). * *P* < 0.05, ** *P* < 0.01

According to the echocardiogram (Fig. 5A–F), compared with the AMI + DMSO group, the AMI + DMSO + ACU group had significantly increased left ventricular anterior wall thickness, along with significantly elevated LVEF and LVFS values. Meanwhile, the percentage of myocardial ischemia area was significantly reduced, and serum NE levels were significantly decreased, which demonstrated the therapeutic effect on AMI. In contrast, compared with the AMI + DMSO + ACU group, the AMI + CPZ + ACU group presented thinner left ventricular anterior wall thickness, decreased LVEF and LVFS values, an increased percentage of myocardial ischemia area, while serum NE levels showed no significant changes. These findings indicate that acupoint injection of TRPV1 inhibitors can reverse the beneficial effects of acupuncture on cardiac function and myocardial ischemia area in AMI rats, but does not affect acupuncture-induced suppression of sympathetic nerve excitability.

## Discussion

Acupoints are the initial sites where acupuncture effects occur and regulate subsequent acupuncture effects. Exploring the mechanisms of acupuncture action from the local acupoint perspective can help understand the common characteristics of acupuncture therapy and provide insights for innovative acupoint stimulation methods. In this study, we compared the effects of acupuncture on the subcutaneous connective layer and the muscle layer on improving cardiac function in AMI rats and found that acupuncture on the muscle layer had a superior effect. Subsequently, it was found that acupuncture at PC6 can upregulate the protein expression of TRPV1 and activate TRPV1 neurons in the DRG. However, when acupuncture was performed after injecting the muscle relaxant succinylcholine (Scc) at the acupoint, it was observed that the activation of the TRPV1 pathway was weakened and the acupuncture effect was lost. Further, after injecting a TRPV1 inhibitor CPZ into the muscle layer at the acupoint and performing acupuncture, the acupuncture effect was also lost. This indicates that the normal contraction function of skeletal muscle at the acupoint is a necessary condition for needle stimulation to activate the TRPV1 channel in the acupoint area. This process mediates the effect of PC6 acupuncture on improving cardiac function in AMI rats and is one of the mechanisms of acupuncture point activation.

As is well known, the structure of acupoints is three-dimensional, comprising the skin, subcutaneous fascia, muscles, nerves, blood vessels, and associated cells[37]. Skeletal muscle is an important component of acupoints, with 55% of acupoints located in regions with thick skeletal muscle[38]. During acupuncture, inducing a local sensation of numbness, heaviness, and distension at the acupoint is referred to as “deqi,” which is crucial for achieving therapeutic effects[39]. Lu Fengyan's research demonstrated that acupuncture into the muscle layer induces a more pronounced deqi sensation compared to acupuncture into the subcutaneous layer [40]. Given that deqi is critical for therapeutic efficacy, this raises an important question: whether the effects of acupuncture into the muscle layer is prior to acupuncture into the subcutaneous connective tissue layer. Therefore, in this study, we compared the therapeutic effects of acupuncture at different tissue layers (subcutaneous layer and muscle layer) in AMI rats. Data from echocardiographic assessments and quantification of myocardial ischemia area in rats showed that both tissue layers improved cardiac function in AMI rats to varying degrees, with acupuncture at muscle layer exhibiting a more significant improvement, which indicating that the muscle layer of the acupoint is the key layer mediating the acupuncture effect.

Contraction is the fundamental and core function of muscles. Whether it is skeletal muscle, cardiac muscle, or smooth muscle, one of the main physiological characteristics of these muscle types is the ability to generate force or movement through contraction, thereby realizing a variety of physiological functions[41]. The myoelectric signals induced by the "deqi" sensation, an important subjective experience in acupuncture and a key factor for its therapeutic efficacy, during acupuncture indicate that acupuncture elicits muscle contraction[10, 42]. A critical question arises: if muscle contraction function is impaired prior to acupuncture, will this affect acupuncture efficacy? To address this question, we used the muscle relaxant Scc to relax the skeletal muscle at PC6 in rats. When acupuncture was performed under this muscle-relaxed condition, we found that its effect on improving the cardiac function of AMI rats was completely abrogated. This result suggests that normal skeletal muscle contraction in the acupoint area is a key factor mediating acupuncture effect. This is the first study to investigate the relationship between the contractile function of local muscles at acupoints and the acupuncture effect. Concurrently, the activation of TRPV1-positive neurons in C5-T1 DRG was examined, and it was observed that acupuncture increased the activation of neurons expressing TRPV1. The DRG is the location where the cell bodies of somatosensory neurons are housed, while the site of acupuncture stimulation is located at peripheral nerve endings. Thus, the activation of DRG neurons indicates that peripheral nerve endings are activated by acupuncture stimulation. These findings indicates that TRPV1 ion channels in the acupoint area are activated by acupuncture targeting the muscle layer. The WB results also indicate increased TRPV1 expression in the acupoint area. Consistent with this, an increase in TRPV1 protein expression in the acupoint area was revealed by western blot (WB) analysis. Pretreatment with Scc at the acupoint prior to acupuncture significantly reduced the activation of TRPV1-expressing neurons in the DRG. This observation confirms that normal skeletal muscle contraction is a necessary prerequisite for acupuncture-induced TRPV1 activation—even acupuncture cannot effectively activate TRPV1 under muscle relaxation.

To further verify the core role of TRPV1 in the acupoint muscle layer, a follow-up experiment was conducted to explore the effect of regulating TRPV1 in this region on the therapeutic effect of acupuncture in AMI rats. It was found that microinjection of the TRPV1 inhibitor CPZ into the muscle layer of the acupoint area could partially reverse the improvement of cardiac function in AMI rats induced by acupuncture. This result suggests that TRPV1 in the muscle layer of the acupoint area may be one of the key factors in triggering the therapeutic effects of acupuncture.

TRPV1 is a multisensory ion channel, initially identified as heat-sensitive and subsequently found to respond to chemical stimuli; it is also thought to be activated by mechanosensitive ion channels[13]. TRPV1 is predominantly expressed at the terminals of nerve fibers and is additionally expressed in various cell types[14], including smooth muscle cells, epithelial cells, vascular endothelial cells, and inflammatory cells[43]. Compared with acupuncture alone, acupuncture at the acupoint following Scc administration resulted in significantly reduced TRPV1 activation, verifying that normal muscle contractility is an important prerequisite for acupuncture to promote TRPV1 activation. Mechanistically, appropriate contraction of the acupoint-region skeletal muscle evoked by acupuncture increases friction between the acupuncture needle and the surrounding skeletal muscle. During needle twisting or lifting-thrusting manipulations (key manual techniques in acupuncture), mechanical forces (e.g., traction and compression) exerted on the surrounding tissues are amplified. Simultaneously, muscle contraction triggers the release of several chemical molecules that enhance the activation of TRPV1 channels, most notably ATP and H⁺. Research indicates that muscle contraction can induce skeletal muscle cells to release ATP [44]. The released ATP acts directly on nociceptive nerve endings and binds to the intracellular ankyrin repeat domain (ARD) of TRPV1, acting as a positive allosteric modulator that lowers the activation threshold of the TRPV1 channel for its cognate ligands [45]. In addition, ATP can activate P2X3 receptors located on nerve endings, thereby initiating the downstream Gq-PLC-PKC signaling cascade. This pathway phosphorylates the TRPV1 channel and sensitizes it to mechanical and chemical stimuli[46–48]. Furthermore, muscle contraction also leads to local hydrogen ion (H⁺) release, resulting in decreased interstitial fluid pH[49]. An acidic environment constitutes a classic endogenous activation condition for TRPV1. Previous studies have confirmed that H⁺ ions can directly interact with specific amino acid residues within the pore region of the TRPV1 channel, thereby significantly increasing its open probability[50]. Based on these findings, we speculate that this cascade may represent the mechanism by which acupuncture-induced muscle contraction promotes TRPV1 channel activation.

In the present study, we did not measure the levels of ATP and H⁺ in the acupoint regions, therefore, the above mechanism remains to be fully confirmed. In future studies, we will perform in vivo experiments to verify whether acupuncture induces muscle contraction and subsequently triggers the release of ATP and H⁺ by measuring ATP and H⁺ concentrations in acupoint regions before and after acupuncture, as well as before and after muscle relaxant administration. Further in vitro experiments will be conducted to isolate DRG neurons and investigate the effects of ATP/H⁺ on calcium influx in these neurons, so as to determine whether ATP/H⁺ activates TRPV1 in DRG neurons. Finally, using gene knockout or knockdown strategies combined with exogenous ATP and H⁺ perfusion, or performing acupuncture intervention coupled with ATP degradation and H⁺ neutralization, we will examine their effects on TRPV1-positive neuron activation in the DRG and acupuncture-related behavioral responses. Collectively, these experiments will clarify whether acupuncture-evoked muscle contraction promotes TRPV1 activation through ATP and H⁺-mediated signaling pathways.

During myocardial ischemia, sympathetic nervous activity is upregulated as a compensatory mechanism, leading to cardiac electrical conduction disturbances[51]. This excitatory state is characterized by sustained elevation in sympathetic tone throughout the progression of myocardial ischemia and heart failure[52]. Acupuncture at PC6 has been shown to improve cardiac function in AMI, which is linked to the inhibition of sympathetic hyperactivity[53–55]. Consistent with this mechanism, our study found that PC6 acupuncture reduced serum norepinephrine levels in AMI rats.

PC6 is anatomically located on the palmar side of the wrist (2 inches proximal to the distal transverse crease, between the palmaris longus and radial wrist flexor tendons)[32]. In rats, the muscles underlying PC6 (extensor radial wrist, extensor ulnar wrist, and common extensor digitorum) are innervated by the median nerve, whose fibers originate from spinal cord segments C5–T1[56]. Following acupuncture-evoked muscle contraction and subsequent activation of TRPV1-expressing neurons in the C5- T1 DRG, the afferent signals first transmit to the spinal dorsal horn, then ascend to the brainstem nuclei (including the nucleus tractus solitarius [NTS] and rostral ventrolateral medulla [RVLM]) and hypothalamic nuclei, thereby rebalancing the sympathetic and parasympathetic tone[57]. A recent study[58] demonstrated that the corticothalamic circuit also participates in the modulation of post-myocardial infarction sympathetic neural function elicited by electroacupuncture at HT7, an acupoint in close anatomical proximity to PC6. Furthermore, cardiac sympathetic preganglionic neurons are distributed within spinal segments C8–T6, which shows partial overlap with the spinal segments innervating PC6. Therefore, acupuncture-evoked afferent signals may directly regulate cardiac autonomic nervous activity via signal transduction within the spinal cord[59]. However, this mechanism still lacks direct confirmatory evidence.

To address the scientific question of whether TRPV1 channels in the acupoint area mediate acupuncture effects, a multitude of relevant investigations have been conducted previously: in mice with complete Freund’s adjuvant (CFA)-induced inflammatory pain, TRPV1 knockout was shown to attenuate the analgesic effect of acupuncture[21]; meanwhile, other studies demonstrated that the injection of TRPV1 agonists into acupoints could mimic such an analgesic effect [22, 23, 60]. These studies, which have focused primarily on acupuncture-induced analgesia, collectively indicate that TRPV1 in the acupoint area serves as a key mediator of acupuncture effects. In the present study, using rats with AMI as experimental subjects, we further confirmed that TRPV1 expressed in the skeletal muscle of acupoints exerts a core mediating role in the cardioprotective effects of acupuncture. Based on the aforementioned studies, we can infer that acupuncture-induced activation of TRPV1 in the acupoint area constitutes an initiating mechanism underlying the therapeutic effects of acupuncture, and this mechanism exhibits a certain degree of universality—at least in acupuncture-mediated analgesia and myocardial protection.

This study has several limitations. First, although the present study clarifies that TRPV1 channels in the muscular layer of the PC6 acupoint region mediate the amelioration of cardiac function in AMI following acupuncture stimulation, the upstream and downstream regulatory networks of TRPV1 remain to be elucidated. 880Third, our results demonstrates that muscle layer-targeted acupuncture yields superior efficacy compared with fascia layer acupuncture in ameliorating AMI-associated cardiac impairment, which may provide reference regarding acupuncture depth for the clinical acupuncture treatment of this condition. However, our standardized acupuncture protocol (administered once daily for seven consecutive days) did not explore the effects of varying stimulation parameters (e.g., frequency, duration, and different acupuncture manipulations) on therapeutic outcomes. This limitation restricts the direct translatability of the current findings to individualized clinical acupuncture regimens. Furthermore, this study only assessed short-term therapeutic effects within a 7-day observation window and did not evaluate the impact of acupuncture on long-term cardiac function recovery, myocardial fibrosis degree, survival rates, or the incidence of AMI-related complications (e.g., cardiac arrhythmias) in AMI rats. Consequently, the long-term stability and durability of the observed therapeutic effects, as well as their suitability for clinical translation, remain undetermined.

Future studies will further elucidate the upstream and downstream signaling pathways of TRPV1, which will help clarify how acupuncture signals are initiated locally at acupoints and then improve cardiac function through the nervous system. Parametric optimization studies will be conducted to explore how different combinations of stimulation frequency and needle retention time modulate TRPV1 activation and the amelioration of cardiac function, thereby identifying optimal acupuncture parameter regimens for AMI. Additionally, extended observation periods will be adopted to systematically evaluate the long-term therapeutic effects of acupuncture.

## Conclusion

**Fig. 6:**
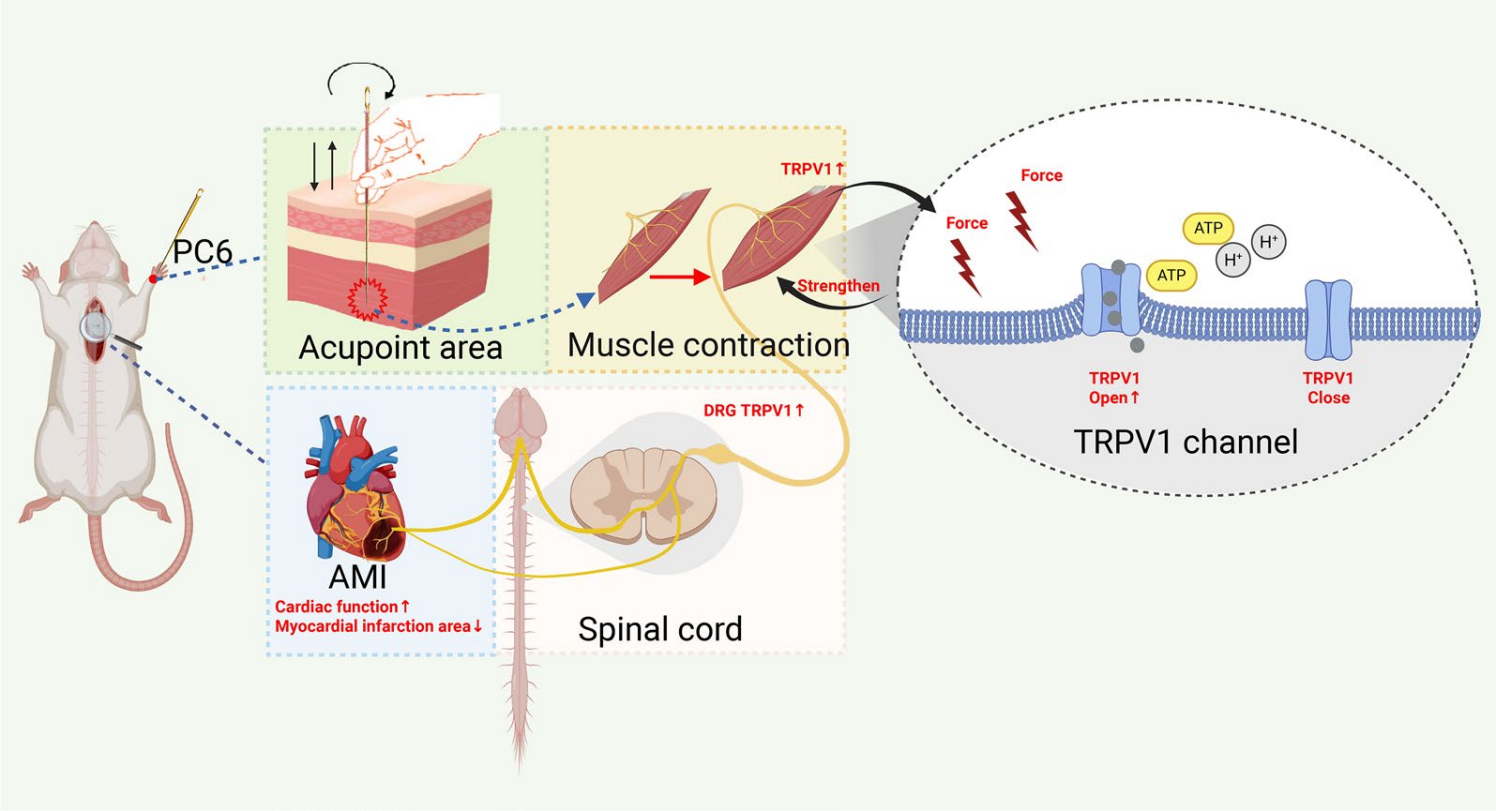
Activation mechanism of acupuncture effects mediated by TRPV1 in the acupoint muscle layer (Created with BioRender.com)

In conclusion, the current study demonstrates that acupuncture targeting the muscle layer of the PC6 (Neiguan) acupoint exerts a more potent effect on improving cardiac function in AMI model rats than acupuncture targeting the subcutaneous layer of the same acupoint. Mechanistically, this study identifies two key factors mediating the cardioprotective effect of acupuncture: first, stimulation of TRPV1 channels in the acupoint muscle layer is a core mediator of acupuncture’s efficacy; second, normal contractile function of the acupoint skeletal muscle is an essential prerequisite for this TRPV1 activation. Our findings provide experimental support for elucidating the mechanism by which TRPV1 in the acupoint muscle layer mediates acupoint activation (Fig. 6). Furthermore, when combined with existing research on TRPV1-mediated acupuncture effects in inflammatory pain, these results suggest that the acupuncture efficacy mediated by TRPV1 exhibits certain universality across distinct disease models, at least in acupuncture-induced analgesia and myocardial protection.

## Supplementary Information

Additional file1

## Data Availability

The datasets used or analyzed throughout this study are available from the corresponding author upon reasonable request.

## Abbreviations

TRPV1: Transient receptor potential vanilloid 1
CFA: Complete Freund’s Adjuvant
AMI: Acute myocardial infarction
LVEF: Left ventricular ejection fraction
EAP: Electroacupuncture pretreatment
MIRI: Myocardial ischemia–reperfusion injury ()
SD: Sprague–Dawley
TTC: 2,3,5-Triphenyltetrazolium chloride
NE: Norepinephrine
ELISA: Enzyme linked immunosorbent assay
Scc: Succinylcholine chloride
DRG: Dorsal root ganglia
IF: Immunofluorescence
WB: Western blotting
CPZ: Capsazepine
