# Supplementary material for: TRPV1 in skeletal muscles mediates the improvement of cardiac function induced by acupuncture at the PC6 acupoint in rats with acute myocardial infarction
Source: Chin Med. 2026 May 6;21:125. doi: 10.1186/s13020-026-01384-2 (PMC13147612; doi:10.1186/s13020-026-01384-2)
Supplement: Supplementary file 1 — Additional file1 [file 13020_2026_1384_MOESM1_ESM.docx]

## Additional file 1

According to echocardiography (Fig.S A), 24 hours post-modeling, rats in the Sham+Vehicle and Sham+Scc groups exhibited thickened left ventricular walls with normal systolic and diastolic contractility. Compared with Sham+Vehicle, the AMI+Vehicle and AMI+Scc groups showed marked thinning of the left ventricular anterior wall and impaired systolic and diastolic function. Based on echocardiographic findings (Fig.S B-C) and TTC staining results (Fig.S D-E), after 7 days of intervention, compared with the Sham+Vehicle group, the Sham+Scc group showed no statistically significant differences in LVEF, LVFS, or myocardial ischemia area. Compared with the AMI+Vehicle group, the AMI+Scc group showed no statistically significant differences in LVEF, LVFS, or myocardial ischemia area. This indicates that the intramyocardial injection of the Scc has no effect on cardiac function in the AMI model rats.


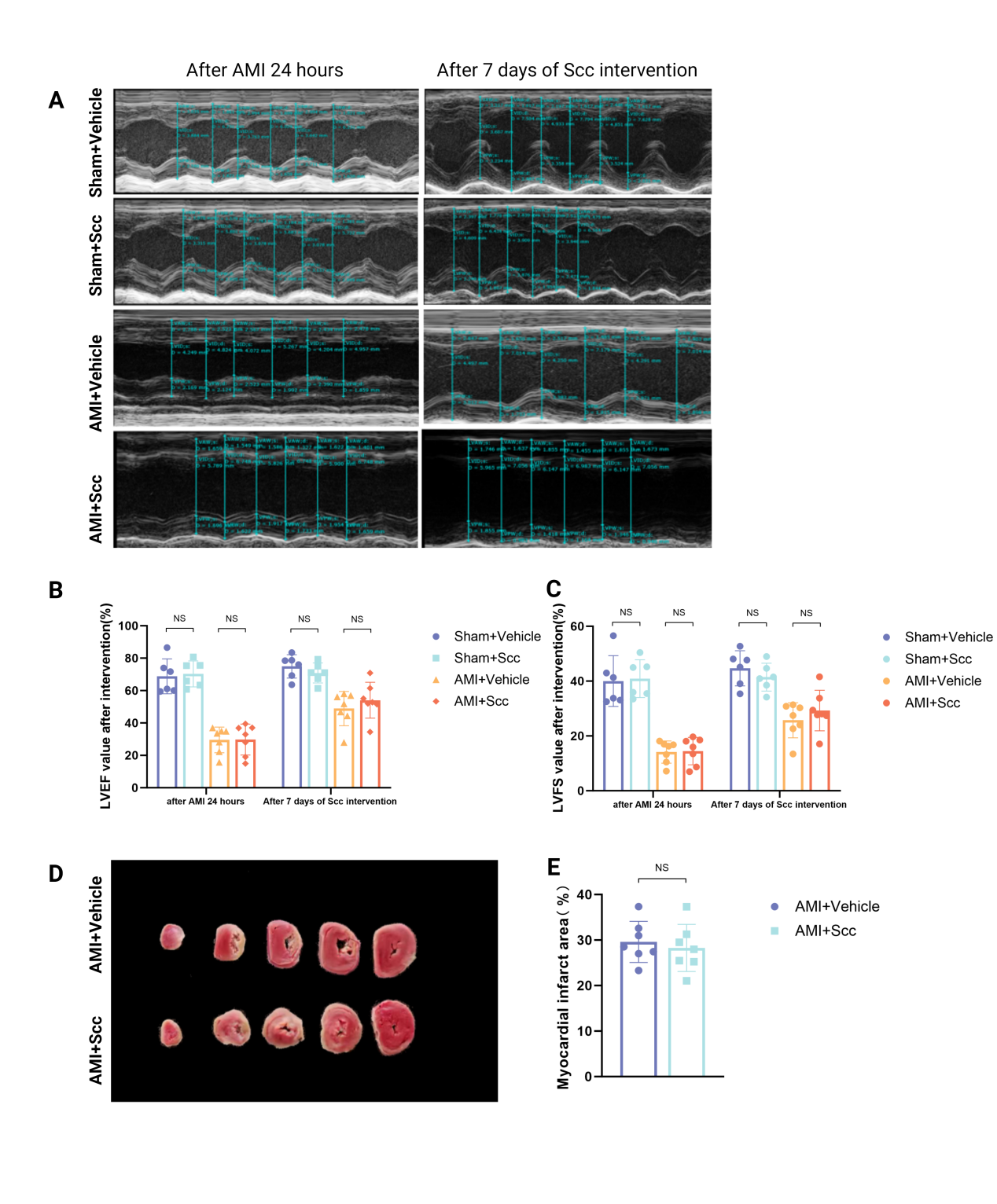


**Fig.S** PC6 injection of Scc does not affect cardiac function in AMI rats. **A** Echocardiography of rat heart. **B** Effect of PC6 injection of Scc on LVEF values in rats.(n=6-7) **C** Effect of PC6 injection of Scc on LVFS values in rats.(n=6-7) **D** Myocardial ischemia area in rats. **E** Effect of PC6 injection of Scc on myocardial ischemia area in rats with AMI.(n=7). * P < 0.05, ** P < 0.01.
